# Effect of *Phlomis persica* on glucose levels and hepatic enzymatic antioxidants in streptozotocin-induced diabetic rats

**DOI:** 10.4103/0973-1296.66940

**Published:** 2010

**Authors:** Parisa Sarkhail, Mohammad Abdollahi, Sedigheh Fadayevatan, Abbas Shafiee, Azadeh Mohammadirad, Gholamreza Dehghan, Hadi Esmaily, Gholamreza Amin

**Affiliations:** Pharmaceutical Sciences Research Center, Tehran University of Medical Sciences, Tehran, Iran; 1 Department of Botany, Faculty of Natural Science, University of Tabriz, Tabriz, Iran

**Keywords:** Antidiabetic, diabetes rats, oxidative stress, *Phlomis persica*, streptozotocin

## Abstract

Methanol extract of the aerial parts of *Phlomis persica* Boiss. (Lamiaceae) (PPE) was studied to evaluate the effects of antidiabetic potential, by measuring fasting blood glucose, insulin, total antioxidant power (TAP), using ferric reducing antioxidant power (FRAP), lipid peroxidation (using thiobarbituric acid reactive substances, TBARS), superoxide dismutase (SOD), catalase (CAT), and glutathione peroxidase (GPx) on streptozotocin-induced diabetes in rats. Male Wistar rats were randomly divided into five groups of six animals each. Oral administration of PPE at doses of 100 and 200 mg/kg once a day for 10 days resulted in a significant reduction in fasting blood glucose and an increase in serum insulin levels, in comparison with diabetic control group. It also prevented diabetes-induced loss in body weight. Hepatic TAP increased and TBARS decreased following PPE treatments. The extract at 100 and 200 mg/kg increased the activity of hepatic SOD, CAT, and GPx in diabetic rats. It is concluded that PPE has antidiabetic potential that is comparable with glibenclamide. In conclusion, the results of the present study show positive effects of *P. persica* on experimental diabetes and thus the antidiabetic effect of PPE is related to its potential to inhibit hepatocellular oxidative stress.

## INTRODUCTION

Type II diabetes mellitus is commonly known as non-insulin dependent diabetes, and is characterized by hyperglycemia and deficiency of secretion or action of endogenous insulin and associated with a number of vascular and neuropathic complications. In recent years, role of oxidative stress as a cellular mechanism in the pathology of diabetes has been described.[1–3] Oxidative stress is involved in the pathogenesis of both types of diabetes mellitus by generation of oxygen free radicals due to nonenzymatic protein glycosylation, auto-oxidation of glucose and also by changing the content of antioxidant defense enzyme.[1,4–7] As diabetes is one of the chronic diseases that is increasing rapidly in the world population, it seems that the screening of new natural sources including plant extracts or compounds for antidiabetic properties is necessary. For many years, various medicinal plants have been used by people in different countries to treat or alleviate diabetes mellitus symptoms and currently studies at basic and clinical levels conducted worldwide have shown these beneficial effects.[8,9] Mechanism of hypoglycemic effect of most of the natural antidiabetic alternatives is thought to be related to the existence of some compounds which stimulate insulin secretion from pancreatic β-cells or possess antioxidant activities.[3,8–10]

A number of *Phlomis* species (Lamiaceae) have been used in folk medicine as stimulants, anticough agents, and to treat gastric, intestinal and abdominal pains, as a tonic, sedative, carminative and astringent.[11] Various activities such as antinociceptive,[12,13] antigenotoxic and antioxidant,[14] antiulcerogenic, anticancer, anti-inflammatory and antiallergic activities have been reported for some *Phlomis* species.[11] This genus is rich in terpenoids, iridoids, flavonoids and other phenolic compounds that contribute potential biological effects like antioxidant activities to many of them. Many of the reports published focused on antioxidant and antimicrobial properties, and the antidiabetic activity of this genus has not been sufficiently investigated.[11,15] A recent study on *Phlomis anisodonta* showed powerful antidiabetic and antioxidant effects in diabetic rats.[16] Latest studies on the alcoholic extract of aerial parts of *Phlomis persica*, which is an endemic species in Iran, showed that it contains terpenoids, iridoids, flavonoids, and other related phenolic compounds[17] and the free radical scavenging activity of the ethyl acetate extract of *P. persica* on DPPH free radicals may be related to its high phenolic content.[18]

Injection of streptozotocin (STZ) in one of the animal models produces diabetes (types I and II diabetes mellitus) by destroying pancreatic β-cells, probably via a free radical mechanism.[19] The purpose of this study was to investigate the effects of *P. persica* methanol extract (PPE) on STZ-induced diabetes in rats, by measuring fasting blood glucose, insulin levels, total antioxidant power (TAP), and hepatocellular lipid peroxidation, and the activities of superoxide dismutase (SOD), catalase (CAT) and glutathione peroxidase (GPx).

## MATERIALS AND METHODS

### Chemicals

Glibenclamide from Tehran Chemistry (Tehran, Iran), STZ from Pharmacia and Upjohn (USA), sodium acetate, ethylenediamine tetraacetic acid (EDTA), FeCl_3_.6H_2_O, sodium sulfate, FeSO_4_, potassium dihydrogen phosphate (KH_2_PO_4_), potassium hydrogen diphosphate (K_2_HPO_4_), 2-thiobarbituric acid (TBA), bovine serum albumin (BSA) from Merck (Iran), 2,4,6-tripyridyl-s-triazine (TPTZ), Folin-Ciocalteu reagent, 1,1,3,3-tetramethoxypropan, xanthine, xanthine oxidase (1 U/mg protein from buttermilk), SOD (4400 U/mg protein, from *Escherichia coli*), GSH, GPx, NADPH, glutathione reductase, NaN_3_ from Sigma-Aldrich (England), and nitroblue tetrazolium (NBT) from Acros Organics (USA) were used in this study. Solvents used were of the highest commercial grade.

### Plant materials and extraction procedures

Aerial parts of *P. persica* (Goosh bareh in Persian) were collected from northeast of Iran (Khorassan province) during the flowering stages. They were identified by Dr. G. Amin and deposited at Herbarium of Faculty of Pharmacy (FP), Tehran University of Medical Sciences (TUMS), with voucher specimen no. 6532 TEH. The air-dried powdered parts (580 g) of P. persica were extracted twice with methanol 80% (2 × 2 l) at 45°C in percolator. The combined methanol extracts were evaporated to dryness under reduced pressure to give solid residues (yield 24%). The residue was stored at 4°C and used for subsequent experiments.[17]

### Animals and induction of diabetes

Male Wistar rats weighing 220–270 g were obtained from animal house of FP/TUMS. The rats were housed in an air-conditioned room at 25 ± 1°C with a lighting schedule of 12 h light and 12 h dark cycle. A standard pelleted diet and tap water were supplied *ad libitium*. After fasting for 16 h, the rats were injected intraperitoneally (i.p.) with a single dose of 40 mg/kg of STZ freshly dissolved in normal saline. Diabetes in rats was identified by polydipsia, polyuria and by measuring fasting blood glucose concentrations 48 h after the injection of STZ. Rats with a blood glucose level 250–280 mg/dl were selected for the experiments. All the experiments were performed according to “The Animal Welfare Act” (Act P.L. 99-198) and all ethical manners were considered carefully.

### Experimental design

The animals (*n* = 30) were randomly divided into five groups of six animals each as given below. Glibenclamide and different doses of PPE were administered in aqueous solution (3% v/v Tween 80 in water) once per day, using an intragastric tube. The groupings are as follows:

Group I: normal control (aqueous solution)

Group II: diabetic control (aqueous solution)

Group III: diabetic-PPE (100 mg/kg)

Group IV: diabetic-PPE (200 mg/kg)

Group V: diabetic-glibenclamide (3 mg/kg)[16]

All the treatments continued for 10 days. On the evening of days 1 and 10, all rats were fasted overnight (16 h) and blood was collected from the tail. Blood samples were centrifuged at 4500 *g* for 10 min to obtain serum. On day 10 of the treatment, rats were anesthetized by administration of pentobarbital (55 mg/kg) and laparotomy was performed. Liver was dissected out and rinsed in ice-cold saline to remove the blood and immediately frozen and stored at −80°C for various assays. Before analyzing, the liver was homogenized in 50 mM phosphate buffer solution (pH 7.4) using a tissue homogenizer at 4°C. The homogenates were centrifuged at 15000 *g* for 20 min and the supernatant was used for analyses.

### Analytical procedures

Blood glucose was measured using glucometer (EasyGluco, infopia Co, Ltd, Korea). Insulin was determined by (Enzyme-Linked ImmunoSorbent Assay) ELISA technique using Mercodia Kit (Sweden). The ability of the samples to reduce Fe^3+^ to Fe^2+^ in ferric reducing antioxidant power (FRAP) method was determined[20] as an index of TAP, as described previously by Benzi and Strains. Thiobarbituric acid reactive substances (TBARS) were determined[21] as an index of lipid peroxidation by the method of Satho. SOD was quantified according to the method described by Ukeda *et al*.[22] CAT was determined by monitoring the decomposition of hydrogen peroxide, as described by Aebi.[23] GPx was determined by the method of Paglia and Valentine.[24] Protein was determined by the method of Lowry *et al*.,[25] using bovine serum albumin as standard.

### Statistical analysis

All data are expressed as mean ± SD. Statistical analysis was performed with one way analysis of variance (ANOVA), followed by Tukey *post hoc* test for multiple comparisons and *P* < 0.05 was considered significant.

## RESULTS

### Effects of *P. persica* methanol extract on fasting blood glucose, serum insulin and body weight

Table 1 shows the effects of PPE on fasting blood glucose, serum insulin and body weight in normal and experimental rats. There was a significant elevation in blood glucose 48 h after administration of STZ. After 10 days, no significant change in blood glucose was noted in normal rats (Group I), while there was a significant elevation in blood glucose and decrease in serum insulin levels in STZ-induced diabetic rats (Group II). The administration of PPE (Groups III and IV) and glibenclamide (Group V) significantly decreased blood glucose and significantly increased serum insulin levels in diabetic rats as compared with diabetic control rats. The decrease in body weight in PPE-treated and glibenclamide-treated groups was significantly less than that of diabetic control rats.

**Table 1:** Effect of PPE on rat blood glucose and insulin levels, and body weight

Group	Glucose (mg/dl)		Insulin (pmol/l)	Change in body weight (g)
	Initial	Final
Normal	63.80 ± 5.60	62.17 ± 7.44	192.10 ± 8.42	63.67 ± 9.58
Diabetic control	258.17 ± 11.32	295.00 ± 20.49*	49.59 ± 7.66*	−72.50 ± 12.77*
Diabetic + PPE (100 mg/kg)	262.33 ± 14.92	76.00 ± 3.58*	127.71 ± 3.19*	−19.33 ± 3.39*
Diabetic + PPE (200 mg/kg)	250.83 ± 8.23	75.00 ± 8.07*	133.66 ± 1.49*	−17.00 ± 3.29*
Diabetic + glibenclamide	259.67 ± 7.12	76.83 ± 6.62*	138.32 ± 9.80*	−27.33 ± 5.57*

Values are given as the mean ± SD for groups of six animals in each.

* Values are statistically significant at *P* < 0.05.

Diabetic control rats were compared with normal rats, PPE-treated diabetic rats were compared with diabetic control, glibenclamide-treated diabetic rats were compared with diabetic control. PPE, *P. persica* methanolic extract

### Effects of *P. persica* methanol extract on liver total antioxidant power

Table 2 summarizes the effects of PPE on liver TAP and lipid peroxidation. There was a significant decrease in TAP of STZ-induced diabetic rats as compared with normal rats. PPE (100 and 200 mg/kg) and glibenclamide treatments significantly increased TAP in diabetic rats, as compared with diabetic control rats. Additionally, doses of 100 and 200 mg/kg of PPE were more effective than glibenclamide in improving TAP of diabetic rats.

**Table 2:** Effect of PPE on rat liver TAP and TBARS

Group	TAP (nmol/mg protein)	TBARS (nmol/mg protein)
Normal	5.22 ± 0.18	0.69 ± 0.01
Diabetic control	1.17 ± 0.09*	2.32 ± 0.21*
Diabetic + PPE (100 mg/kg)	3.64 ± 0.49*	0.79 ± 0.02*
Diabetic + PPE (200 mg/kg)	3.69 ± 0.01*	0.82 ± 0.04*
Diabetic + glibenclamide	2.01 ± 0.16*	0.72 ± 0.04*

Values are given as mean ± SD for groups of six animals in each.

* Values are statistically significant at *P* < 0.05.

Diabetic control rats were compared with normal rats, PPE-treated diabetic rats were compared with diabetic control, glibenclamide-treated diabetic rats were compared with diabetic control. PPE, *P. persica* methanolic extract

### Effects of *P. persica* methanol extract on liver lipid peroxidation

There was a significant elevation in tissue TBARS in diabetic rats as compared with normal rats. Administration of PPE (100 and 200 mg/kg) and glibenclamide significantly decreased lipid peroxidation in liver of diabetic rats and the levels reached near to normal values.

### Effects of *P. persica* methanol extract on liver superoxide dismutase, catalase and glutathione peroxidase

Table 3 shows the effects of PPE on liver SOD, CAT and GPx. During diabetes, there was a significant reduction in the activities of SOD, CAT and GPx. PPE (100 and 200 mg/kg) and glibenclamide treatments significantly increased SOD as compared with diabetic control group. Additionally, the effect of PPE (100 and 200 mg/kg) was significantly greater than that of glibenclamide. There was a significant increase in CAT of diabetic rats treated with doses of 100 and 200 mg/kg of PPE and glibenclamide when compared with diabetic control rats. GPx significantly increased in the groups of rats treated with doses of PPE (100 and 200 mg/kg), and glibenclamide as compared with diabetic control rats.

**Table 3:** Effect of PPE on rat liver SOD, CAT and GPx activities

Group	SOD (U^a^/mg protein)	CAT (U^b^/mg protein)	GPx (U^c^/mg protein)
Normal	33.60 ± 3.19	74.10 ± 3.26	41.92 ± 2.11
Diabetic control	7.86 ± 1.18*	11.18 ± 1.28*	9.40 ± 1.72*
Diabetic + PPE (100 mg/kg)	21.32 ± 0.65*	35.02 ± 0.76	26.90 ± 1.38*
Diabetic + PPE (200 mg/kg)	23.92 ± 0.21*	41.0 ± 3.44*	26.72 ± 0.78*
Diabetic + glibenclamide	15.90 ± 1.03*	50.3 ± 5.78*	21.71 ± 1.15*

Values are given as the mean ± SD for groups of six animals in each. Values are statistically significant at

* *P* < 0.05. Diabetic control rats were compared with normal rats, PPE-treated diabetic rats were compared with diabetic control, glibenclamide-treated diabetic rats were compared with diabetic control. PPE, *P. persica* methanol extract,

a One unit of SOD is defined as the amount of enzyme required to inhibit the rate of NBT reduction by 50%;

b one unit of CAT is defined as millimoles of H_2_O_2_ decomposed/min;

c one unit of GP× is defined as 1 μmol NADPH oxidized/min

## DISCUSSION

Type II diabetes is an endocrine dysfunction that is characterized by chronic hyperglycemia and decrease of insulin secretion or incapability of the peripheral tissues to respond to insulin and is usually associated with a loss of weight.[26]Animal models of non-insulin dependent diabetes (type II diabetes) could be produced after administering a single mild dose of STZ (40 mg/kg) in the adult rats.[19,27]

This study demonstrated that administration of PPE for 10 days reduces fasting blood glucose and increases the level of insulin in STZ-induced diabetic rats. Moreover, the weight loss recovered by PPE treatment. The present data indicate that glibenclamide reduces blood glucose and increases insulin levels in diabetes, which is consistent with previous studies.[16,28] The possible mechanism by which PPE brings its anti-hyperglycemic action may be through membrane depolarization and stimulation of Ca^2+^ channels influx, like glibenclamide, leading to release of more insulin as recommended by other researchers too.[29,30]Supporting this finding, there are numerous studies showing hypoglycemic effects for some plants that contain iridoids, flavonoids and related phenolic compounds.[8,31,32] Furthermore, there is evidence showing that some terpenoids and flavonoids stimulate insulin secretion from pancreatic β-cells, possibly by blocking ATP-sensitive potassium and L-type Ca^2+^ channels on pancreatic β-cells[10] like glibenclamide,[33] activation of the cAMP/PKA signaling,[34] and antioxidant activities.[3] On the other hand, some flavonoids have been found to inhibit glucose transporters in the intestine,[35] increase the storage of glucose in the liver (up-regulated glycogenesis) and reduce glycogen breakdown.[36]

Phytochemical study of PPE has shown that it contains terpenoids, iridoids, flavonoids and other phenolic compounds.[17,18] These components may then be responsible for antihyperglycemic effect of PPE observed in the present investigation. Loss of body weight has been related to diabetes mellitus. Observations of this study showed that the PPE-treated and glibenclamide-treated groups significantly ameliorated the weight loss than that of diabetic control rats.

The increase in oxygen free radicals in diabetes could be related to rise in blood glucose levels, leading to auto-oxidation to generate free radicals.[1,9] Increased concentration of TBARS and decreased level of TAP were observed in liver tissue during diabetes. In this study, hepatic TAP significantly increased and TBARS decreased following PPE treatment, similar to glibenclamide-treated group. Lipid peroxide-mediated tissue damage has been observed in the development of diabetes mellitus. Elevated level of lipid peroxidation in tissues of STZ-induced diabetic rats is one of the characteristic features of chronic diabetes.[30,33] In diabetes, it is thought that hypoinsulinemia increases the activity of the enzyme, fatty acyl coenzyme A oxidase, which initiates β-oxidation of fatty acids, resulting in lipid peroxidation. The increase of lipid peroxidation levels causes functional impairment of membrane by decreasing membrane fluidity and through changing the activity of membrane-bound enzymes and receptors.[37] Lipid peroxidation will in turn result in elevated production of free radicals that are harmful to cells in the body.[3]

The present data also show that STZ-induced diabetes disturbs actions of hepatic antioxidant enzymes (SOD, CAT and GPx). The decreased activities of SOD, CAT and GPx in liver during diabetes mellitus may be due to the production of reactive oxygen free radicals that can themselves reduce the activity of these enzymes.[33,38] These enzymes could destroy the peroxides and play a significant role in providing antioxidant defenses to an organism. In the enzymatic antioxidant defense system, SOD and CAT are the two important scavenging enzymes that remove superoxide radicals (O_2_^.^) and hydrogen peroxide, respectively, *in vivo*. Decrease in GPx activity was also observed in tissues during diabetes. GPx plays a main role in minimizing oxidative damage and is known to be involved in the elimination of low H_2_O_2_ concentrations, whereas CATissensitive to higher concentrations of H_2_O_2_.[6,38,39] Decrease in SOD, CAT and GPx activities may be due to inadequacy of antioxidant defenses in combating reactive oxygen species (ROS) production.[30] The positive effect of PPE on these antioxidant enzymes is most probably due to the existence of iridoid glycosides, flavonoids and other phenolic compounds in PPE,[17,18] as well-known antioxidants[8,40,41] which scavenge the free radicals generated during diabetes.

The present findings support recent report about positive effects of *P. anisodonta* in experimental diabetes[16] and provide some valuable insight into the hypoglycemic potency of *P. persica* in STZ-induced diabetes. In addition, it is concluded that antidiabetic effect of PPE may be related to its potential to inhibit hepatocellular oxidative stress. However, we believe that *P. persica* should be considered as a good candidate like *P. anisodonta* for further investigations to identify the responsible isolated components for antidiabetic effect.

## References

[1] Maritim AC, Sanders RA, Watkins JB (2003). Diabetes, oxidative stress, and antioxidants: A review. J Biochem Mol Toxicol.

[2] Radfar M, Larijani B, Hadjibabaie M, Rajabipour B, Mojtahedi A, Abdollahi M (2005). Effects of pentoxifylline on oxidative stress and levels of EGF and NO in blood of diabetic type-2 patients; a randomized, double-blind placebo-controlled clinical trial. Biomed Pharmacother.

[3] Rahimi R, Nikfar S, Larijani B, Abdollahi M (2005). A review on the role of antioxidants in the management of diabetes and its complications. Biomed Pharmacother.

[4] Osawa T, Kato Y (2005). Protective role of antioxidative food factors in oxidative stress caused by hyperglycemia. Ann N Y Acad Sci.

[5] Vincent AM, Russell JW, Low P, Feldman EL (2004). Oxidative stress in the pathogenesis of diabetic neuropathy. Endocr Rev.

[6] Baynes JW (1991). Role of oxidative stress in development of complications in diabetes. Diabetes.

[7] Larijani B, Afshari M, Astanehi-Asghari F, Mojtahedi A, Rezaie A, Hosseinnezhad A (2006). Effect of short-term carvedilol therapy on salivary and plasma oxidative stress parameters and plasma glucose level in type II diabetes. Therapy.

[8] Li WL, Zheng HC, Bukuru J, De Kimpeb N (2004). Natural medicines used in the traditional Chinese medical system for therapy of diabetes mellitus. J Ethnopharmacol.

[9] Murugan P, Pari L (2006). Antioxidant effect of tetrahydrocurcumin in streptozotocin-nicotinamide induced diabetic rats. Life Sci.

[10] Hoa NK, Norberg A, Sillard R, Van Phan D, Thuan ND, Dzung DT (2007). The possible mechanisms by which phanoside stimulates insulin secretion from rat islets. J Endocrinol.

[11] Limem-Ben Amor I, Boubaker J, Ben Sgaier M, Skandrani I, Bhouri W, Neffati A (2009). Phytochemistry and biological activities of Phlomis species. J Ethnopharmacol.

[12] Sarkhail P, Abdollahi M, Shafiee A (2003). Antinociceptive effect of *Phlomis olivieri* Benth., *Phlomis anisodonta* Boiss. total extracts. Pharmacol Res.

[13] Hajarolasvadi N, Zamani MJ, Sarkhail P, Khorasani R, Mohajer M, Amin G (2006). Comparison of antinociceptive effects of total, water, ethyl acetate, ether, and n-butanol extracts of Phlomis anisodonta Boiss and indomethacin in mice. Intl J Pharmacol.

[14] Dellai A, Mansour HB, Limem I, Bouhlel I, Sghaier MB, Boubaker J (2009). Screening of antimutagenicity via antioxidant activity in different extracts from the flowers of Phlomis crinita Cav. ssp mauritanica munby from the center of Tunisia. Drug Chem Toxicol.

[15] Zhang Y, Wang ZZ (2009). Phenolic composition and antioxidant activities of two Phlomis species: A correlation study. C R Biol.

[16] Sarkhail P, Rahmanipour S, Fadyevatan S, Mohammadirad A, Dehghan G, Amin G (2007). Antidiabetic effect of Phlomis anisodonta: Effects on hepatic cells lipid peroxidation and antioxidant enzymes in experimental diabetes. Pharmacol Res.

[17] Sarkhail P, Monsef-Esfehani HR, Amin G, Surmaghi MH, Shafiee A (2006). Phytochemical study of *Phlomis olivieri* Benth. and *Phlomis persica* Boiss. Daru.

[18] Moein S, Farzami B, Khaghani S, Moein MR, Larijani B (2007). Antioxidant properties and prevention of cell cytotoxicity of *Phlomis persica* Boiss. Daru.

[19] Fröde TS, Medeiros YSJ (2008). Review. Animal models to test drugs with potential antidiabetic activity. Ethnopharmacol.

[20] Benzie IF, Strain JJ (1991). Ferric reducing/antioxidant power assay: Direct measure of total antioxidant activity of biological fluids and modified version for simultaneous measurement of total antioxidant power and ascorbic acid concentration. Meth Enzymol.

[21] Satho K (1978). Serum lipid peroxidation in cerebrovascular disorders determined by a new colorimetric method. Clin Chem Acta.

[22] Ukeda H, Maeda S, Ishii T, Sawamura M (1997). Spectrophotometric assay for superoxide dismutase based on tetrazolium salt 3′–1–(phenylamino)-carbonyl–3, 4-tetrazolium]-bis(4-methoxy-6-nitro)benzenesulfonic acid hydrate reduction by xanthine-xanthine oxidase. Anal Biochem.

[23] Aebi H (1984). Catalase *in vitro* assay. Meth Enzymol.

[24] Paglia DE, Valentine WN (1967). Studies on the quantitative and qualitative characterization of glutathione peroxidase. J Lab Med.

[25] Lowry OH, Roserbrough NJ, Farr AL, Randell RJ (1951). Protein measurement with the folin phenol reagent. J Biol Chem.

[26] Odetola AA, Akinloye O, Egunjobi C, Adekunle WA, Ayoola AO (2006). Possible antidiabetic and antihyperlipidaemic effect of fermented *Parkia biglobosa* (JACQ) extract in alloxan-induced diabetic rats. Clin Exp Pharmacol Physiol.

[27] Lee JJ, Yi HY, Yang JW, Shin JS, Kwon JH, Kim CW (2003). Characterization of streptozotocin- induced diabetic rats and pharmacodynamics of insulin formulations. Biosci Biotechnol Biochem.

[28] Saravanan R, Pari L (2005). Antihyperlipidemic and antiperoxidative effect of Diasulin, a polyherbal formulation in alloxan induced hyperglycemic rats. BMC Complement Altern Med.

[29] Jelodar GA, Maleki M, Motadayen MH, Sirus S (2005). Effect of fenugreek, onion and garlic on blood glucose and histopathology of pancreas of alloxan-induced diabetic rats. Indian J Med Sci.

[30] Pari L, Latha M (2005). Antidiabetic effect of *Scoparia dulcis*: Effect on lipid peroxidation in streptozotocin diabetes. Gen Physiol Biophys.

[31] El Naggar EB, Bartosikova L, Zemlicka M, Svajdlenka E, Rabiskova M, Strnadova V (2005). Antidiabetic effect of *Cleome droserifolia* aerial parts: Lipid peroxidation-induced oxidative stress in diabetic rats. Acta Vet Brno.

[32] Grace MH, Ribnicky DM, Kuhn P, Poulev A, Logendra S, Yousef GG (2009). Hypoglycemic activity of a novel anthocyanin-rich formulation from lowbush blueberry, Vaccinium angustifolium Aiton. Phytomedicine.

[33] Rajasekaran S, Sivagnanam K, Ravi K, Subramanian S (2005). Antioxidant effect of Aloe vera gel extract in streptozotocin-induced diabetes in rats. Pharmacol Rep.

[34] Liu D, Zhen W, Yang Z, Carter JD, Si H, Reynolds KA (2006). Genistein acutely stimulates insulin secretion in pancreatic ²-cells through a cAMP-dependent protein kinase pathway. Diabetes.

[35] Shimizu M, Kobayashi Y, Suzuki M, Satsu H, Miyamoto Y (2000). Regulation of intestinal glucose transport by tea catechins. Biofactors.

[36] Valsa AK, Sudheesh S, Vijayalakshmi NR (1997). Effect of catechin on carbohydrate metabolism. Indian J Biochem Biophys.

[37] Manonmani G, Bhavapriya V, Kalpana S, Govindasamy S, Apparanantham T (2005). Antioxidant activity of *Cassia fistula* (Linn.) flowers in alloxan induced diabetic rats. J Ethnopharmacol.

[38] Kaleem M, Asif M, Ahmed QU, Bano B (2006). Antidiabetic and antioxidant activity of *Annona squamosa* extract in. streptozotocin-induced diabetic rats. Singapore Med J.

[39] Sathishsekar D, Subramanian S (2005). Antioxidant properties of Momordica Charantia (bitter gourd) seeds on Streptozotocin induced diabetic rats. Asia Pac J Clin Nutr.

[40] Delaporte RH, Sanchez GM, Cuellar AC, Giuliani A, Palazzo de Mello JC (2002). Anti-inflammatory activity and lipid peroxidation inhibition of iridoid lamiide isolated from Bouchea fluminensis (Vell.) Mold. (Verbenaceae). J Ethnopharmacol.

[41] Harput US, Calis I, Saracoglu I, Donmez AA, Nagatsu A (2006). Secondary metabolites from *Phlomis syriaca* and their antioxidant activities. Turk J Chem.

